# The risk-value trade-off: price and brand information impact consumers’ intentions to purchase OTC drugs

**DOI:** 10.1186/s40545-020-00293-5

**Published:** 2021-01-25

**Authors:** Lisa Aufegger, Celine Yanar, Ara Darzi, Colin Bicknell

**Affiliations:** NIHR Imperial Patient Safety Translational Research Centre, Imperial College London, London, W2 1PE UK

**Keywords:** OTC advertisement, Brand, Price information, Perceived risk, value, quality, Purchase intention

## Abstract

**Background:**

European countries face fiscal pressure regarding the long-term sustainability of their healthcare system due to increasing levels of public health expenditures and mounting demographic pressures. The promotion of generic drugs is considered to be an efficient means to tackle these challenges; however, market diffusion remains slow. The aim of this study was to investigate the impact of price and brand cues on purchase intentions by means of Direct-to-Consumer (DTC) advertising, and to build on the market cue evaluation model by Dodd et al.

**Methods:**

Participants rated purchase intentions on six DTC adverts varying in price and brand information, followed by self-reports on purchase intentions, attitudes towards generics, brand loyalty, price consciousness, as well as perceptions of quality, risk and value. Open-ended questions explored attitudes toward generic drugs.

**Results:**

Brand information and purchase intentions were mediated by perceived risk and perceived quality, while price information influenced purchase intention through perceptions of quality, risk and value. Consumers’ purchase behaviour was furthermore  influenced by unawareness and misconceptions, past experiences, and advertising as a decision-making tool.

**Conclusions:**

Advertisements, including price and brand information, are an important tool to improve consumers’ awareness of the availability of different OTC drugs. Practical and theoretical implications are discussed.

## Introduction

Increasing needs to curb healthcare cost and growing public demands for self-medication have led to a major expansion of Europe’s Over-the-Counter (OTC) drug market [1]. Within Europe, Germany is the leading market for OTC drugs and is characterised by continuous growth. Between 2017 and 2018 alone the German market witnessed an 3% increase in revenue and is forecasted to depict a profitable growth path over the years ahead [1, 2]. At the same time, Germany, like most other EU member states, faces fiscal pressure regarding the long-term sustainability of its healthcare system, driven by high levels of public expenditure, increased prevalence of chronic disease and mounting demographic pressure [3]. In light of this, many official institutions, both inside and outside the European Union (i.e., the European Parliament, the World Health Organisation, etc.), have emphasised the importance of an active and targeted promotion of responsible self-medication with OTC medicines as a critical building block for effective and efficient healthcare [4].

Generics, in particular, are regarded as the best way for patients to receive similar treatments at lower costs [5]. They enter the market once the patent of a branded drug has expired, allowing for the market to change from a monopoly to oligopoly, where generics and branded drugs compete [6]. The price of generics is considerably lower as manufacturers do not bear the costs of research and development [4], whilst, for quality assurance and marketing authorisation, they must contain the same quantity of active ingredients and must have the same therapeutic effect on an average consumer as its proprietary counterpart [7, 8]. As a consequence, generics appear to constitute an effective tool for increasing self-medication whilst minimising consumers’ and patients’ out-of-pocket costs [9].

Strategies to promote the use of OTC drugs vary across Europe [10], ranging from regulatory reclassification of drugs from prescription-only to OTC status; increasing availability of cheaper non-branded OTC drugs; legal obligation to dispense low-cost generic prescription drugs unless the physician prohibits it; and, the set up computerised prescription order systems that strongly encourage the prescription of generic alternatives. Considering that OTC drugs are excluded from statutory and private health insurance coverage [11], the aim is to reduce healthcare expenditure by transferring certain reimbursement costs from the government to the individual consumer [12]. Research, however, shows that the market diffusion of generic OTC drugs has been slow [6]. This raises the question as to why consumers might hesitate to purchase generics.

### Factors influencing consumers’ purchase intentions

Economic theory suggests that a perfectly rational consumer would choose the least costly product in the pharmaceutical market [6, 13]. Indeed, evidence has shown a considerable shift in preference from expensive to cost-reduced OTC drugs when consumers were informed about the price of the different packages [14], as opposed to consumers choosing the most well-known OTC drug brand when price information was absent. Because the price of generic drugs is generally 10–80% lower than that of a branded OTC drugs, price information may play a decisive role in consumer purchase behaviour [15].

More recent conceptual models argue that consumers do not simply perceive price as the cost of a product but also as a cue for various intervening external and internal constructs, including brand [16, 17], price consciousness [18], and perceived quality, risk and value, respectively [12, 14]. Before generics enter the market, consumers become familiar with a brand through advertising, product displays in pharmacies or prior purchases [19]. According to cue utilisation theory, the experiences with a product contribute to building a brand image, which influences consumers’ evaluation of a product and, in turn, affects their purchase intention [20]. Moreover, past experience and familiarity represent key constituents of brand loyalty [21], as they are often perceived to be of higher quality [22], lower risk [19, 23] and better value for money than unfamiliar products [24]. Since well-known brands are generally highly recognised and considered to hold high credibility [25], consumers may prefer to maintain the status quo by selecting a familiar branded OTC drug at generic entry.

**Fig. 1:**
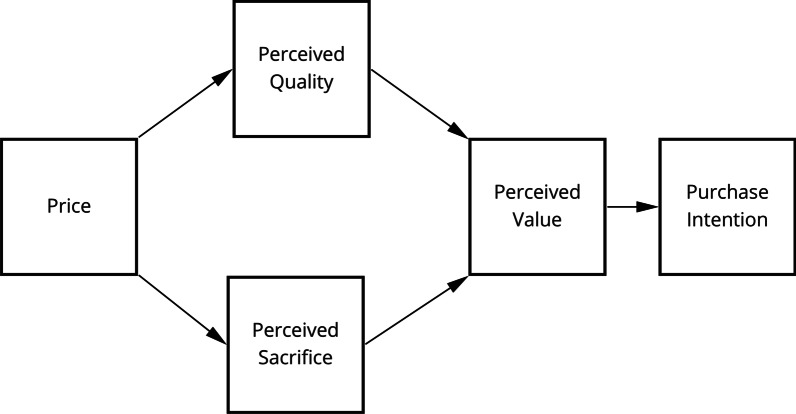
Original market cue-product evaluation model [26]

Lastly, and building on the above, Dodds, Monroe and Grewal’s market cue—product evaluation model [26] assumes a dual role of price information, which has a positive relationship with perceived quality but an inverse relationship with perceived (monetary) sacrifice [27]. Perceived quality refers to ‘...a consumer's judgment about the superiority or excellence of a product’ [28], whereas perceived sacrifices can be defined as ‘...an indicator of the amount of sacrifice needed to purchase a product’ [26]. While the former increases consumers’ purchase intention, the latter is assumed to diminish it (cf. Fig. 1). The cognitive trade-off between these two constructs is considered to be instrumental in the formation of value perceptions, which represents a direct antecedent of consumers’ willingness to buy. Hence, consumers may view products as poor value for money if the perceived sacrifices outweigh perceived quality.

### Context

Empirical findings and theoretical conceptualizations appear to agree that price and brand information reflect important extrinsic cues which exert their influence on purchase intention through a range direct and indirect influencing factors, including brand, brand loyalty, and perceived quality, risk, and value [29].

According to the current state of our knowledge, however, no previous research has aimed to explain the relationship between extrinsic cues in advertising, potentially intervening variables, and their impact on the purchase of OTC drugs [30, 31]. Furthermore, there has been limited conclusive evidence on the impact of both price and brand cues on consumer drug purchasing behaviour [32]. Because research on consumers’ purchase behaviour of OTC drugs has often failed to provide a more integrated and holistic analysis of variables that influence consumers’ decision-making process, an understanding of these variables and their influence on consumer purchase behaviour may help to bridge the gap between the obvious economic benefit of generics and their slow market diffusion.

The present study addressed basic effects of OTC drug advertisements (pain medication), including price and brand cues, on consumers’ purchase intention. Secondly, at a theoretical level, a modified version of Dodd et al.’s market cue evaluation model was tested to explain how price and brand information shapes purchase intentions through a number of intervening constructs, including: brand loyalty; price consciousness; attitude towards generics as well as the products’ perceived risk; and, quality, value and efficacy. Specifically, four main hypotheses are proposed [33] (cf. Table 1).

**Table 1:** Research questions and hypotheses related to purchase intentions of OTC drugs

Research Question: Does price advertising affect consumer purchase patterns of OTC drugs?	
*Hypothesis 1 Participants presented with different OTC advertisements show significantly different purchase intensions*	
H1(a)	a. Participants’ purchase intention is greater for the branded OTC drug compared to the generic OTC drug when no price is presented (brand effect)
H1(b)	b. Participants’ purchase intention is greater for the low-priced generic OTC drug compared to the high-priced branded drug (price awareness)
Research Question: How do relevant perceptual factors affect the relationship between price or brand cues and consumers’ purchase intention?	
*Hypothesis 2 Price and brand information explain a significant proportion of the variance of purchase intention in absence of other potential predictors*	
*Hypothesis 3 Perceived quality and perceived risk fully mediate the relationship between brand information and purchase intention*	
H3(a)	a. Exposure to branded drug advertisements increases perceptions of quality and decreases perceptions of risk, whilst the reverse is true for advertisements of generics
H3(b)	b. There is a significant positive relationship between perceived quality and purchase intention
H3(c)	c. There is a significant inverse relationship between perceived risk and purchase intention
*H4 Perceived quality and perceived risk fully mediate the relationship between price information and perceived value, which in turn predicts purchase intention*	
H4(a)	a. Exposure to high-priced drug advertisements increases perceptions of quality and decreases perceptions of risk (original price-perceived quality effect), the reverse is true for low-priced advertisements
H4(b)	b. There is a significant positive relationship between perceived quality and perceived value
H4(c)	c. There is a significant inverse relationship between perceived risk and perceived value

## Methods

In line with previous literature on (pharmaceutical) advertising and consumer behaviour [34, 35], an experimental design was chosen to explore the possible causative effect of price and brand cues on consumers’ purchase intentions. This type of design is considered the gold-standard for research as it is most rigorous [36]. In comparison to observational or quasi-experimental studies, it offers the greatest control over potential confounding biases and, thus, threats to internal validity [37]. More importantly, it allows researchers to infer direct causal effects if designed appropriately. Since experimental designs are restricted in their explanatory value, a qualitative measure was added to shed further light on consumers’ views on OTC advertising [38]. Although limitations in terms of generalisability should not be disregarded, this design offers a common and effective means to draw conclusions about the effect of extrinsic advertising cues on consumers’ purchase intentions.

### Sample recruitment

An open online questionnaire on Qualtrics was designed run between January 2019 and July 2019. Participants from the German general public were recruited using social media, company mailings and Internet forums. An online advertisement briefly outlining the study aims and procedure was used to invite participants to take part in the online questionnaire. The advert included an embedded link to the questionnaire. Written informed consent was sought from each participant prior their participation. A data protection statement was also provided. Eligible participants were participants aged 18 or over without cognitive or linguistic impairment. Participants received no financial reimbursement and were free to withdraw active involvement in the study at any time of the data collection. All data was anonymised for storage and analysis.

The questionnaire presented participants with one of six pictorial advertisements showing either a well-known OTC brand or a generic alternative. The advertisements were followed by several questions assessing the influence of various factors on participants’ decision-making process. Open-ended questions were added to give participants the opportunity to expand on their response and to gain rich, in-depth information regarding factors influencing participants’ opinion and their decision-making process.

### OTC advertisements

Six pictorial advertisements were created showing either a well-known OTC-brand or a generic alternative. The design of the advertisements was inspired by existing pharmaceutical advertisements and used an identical background and promotional slogan across conditions (”the pill against pain and fever”). As per definition, the generic alternative had the same indications and active pharmaceutical agent varying only in some auxiliary agents. Both drugs are used to treat fever, inflammation, mild or chronic pain and are available in most German pharmacies and online stores.

Four of the six advertisements showed price information, presenting a high or a low price (6.18€ vs. 2.18€). To increase ecological validity, price levels were established by calculating the average price for which each drug is sold in German pharmacies (online and in retail stores). To ensure that participants had the same basic knowledge about the stimuli they were presented with, the terms *Generics*, *OTC-drug* and the active pharmaceutical agent was explained on a separate information sheet. The stimuli and the information sheet were pre-assessed by a group of nine medical marketing experts from an unrelated German pharmaceutical company. Lastly, manipulation checks were embedded which assessed the attention paid to the presented stimuli advertisements. Participants were asked to select whether they saw a generic or a brand, state the name of the drug they saw and either rate the price-level from very low to very high or to write down how much they would pay for the seen drug. The latter question was asked when the advertisement showed no price information.

### Questionnaires

Questionnaires addressed participants’ (1) demographics; (2) brand loyalty; (3) price consciousness; (4) attitude towards generics as well as the products’ perceived (5) risk; and, (6) quality, value and efficacy. The main outcome variable was (7) purchase intention. Except for the demographics, all items were rated on a 7-point Likert Scale (1 = “strongly disagree” to 7 = “strongly agree”), with higher scores indicating higher levels of perceived risk, quality, etc. Lastly, (8) open-ended questions at the end of the survey enquired about general attitudes towards generics as opposed to branded OTC drugs, as well as general comments participants wished to make (i.e. “To what extent does the presentation of prices in adverts would affect whether I buy generic or branded OTC drugs”, and “Do you have any further comments?”).

(1) Demographics

Participants' demographic profile was captured using a multiple-choice response format and asked them to report their age, sex, net income, highest level of education, type of job and health insurance.

(2) Brand loyalty

Eight items developed by Lodorfos et al. (2006) were used to measure participants’ brand loyalty [39]. Example items are ‘I prefer to purchase a brand of pharmaceutical product that I have previously purchased.’ The scale was found to be reliable with *α* = 0.78.

(3) Price consciousness

Price consciousness was measured to investigate the role of individual differences in the utilisation of price cues in consumer purchasing patterns [40]. Price consciousness was assessed including statements such as ‘Price is the most important factor when I am choosing a brand’. In line with a recent study on OTC drugs [41], one item was added ‘The higher the price, the higher the quality of the product’. The scale was found to be reliable (*α* = 0.80).

(4) Attitude towards generics

Attitude towards generics was assessed based on Hakonsen et al. 2018. Example items are ‘I don’t like the thought of generics’ and ‘Generics are often associated with more side-effects than brand-name drugs’ [42].

(5) Perceived risk

Perceived risk was assessed using Stone and Grønhaug nine-item scale of perceived risk dimensions, including performance, physical and financial risk [43]. Internal consistency was 0.91 for perceived performance risk, 0.96 for perceived physical risk and 0.93 for perceived financial risk.

(6) Perceived quality, value and efficacy

Three items on perceived quality indicators and five value indicators were used to measure participants’ perceptions [26, 44]. The latter were only shown to those participants in conditions presented with price information. Efficacy perceptions were assessed, such as ‘In your opinion, if this drug did help a person’s medical condition, how much would it help?’ [45]. All scales have been shown to be robust and reliable with an internal consistency of 0.93 for perceived quality and 0.95 for perceived value, and 0.83 for efficacy.

(7) Purchase Intention

Purchase intention was measured using a four-item Likert scale [26], and included items such as: ‘If I were shopping for an OTC product, the likelihood that I would purchase this brand is high’. The scale has been widely adopted within the previous literature [46–49], and has shown adequate internal consistency (*α* = 0.96). Participants were also asked to rate the following question ‘The presentation of prices in advertisements would affect whether I buy generic or branded OTC drugs.’ on a scale ranging from strongly disagree [1] to strongly agree [7] and to explain their response in a free text box.

(8) Open-ended questions

Lastly, participants were asked to state their general attitudes towards generic drugs in an open-ended text box, as well as encouraged to provide any additional feedback or comments they found important for the research team to consider.

All items were translated into German and revalidated. Following recent guidelines for translating questionnaires, a bilingual researcher and a naïve bilingual translator, who was unaware of the objective of the study, translated the items into their mother tongue to best reflect the nuances of the German language. After the data collection, reliability checks were conducted to assess for internal consistencies.

## Data processing and treatment

Questionnaire responses were securely stored online in a password protected Qualtrics account, which allocated a unique identifier code to the data of each participant. The data was imported into Microsoft Excel and IBM SPSS (Version 25) for further analysis. Participants’ responses to all Likert-scale items, apart from those assessing demographics, were calculated to an average score for each variable. The data was tested for normal distribution before statistical analysis was applied [36]. In the case of assumption violations, the choice of test was adapted to the data accordingly. Prior to the main analysis, participants’ demographic characteristics were explored by looking at the frequencies of their occurrence. Sensitivity-analysis was also conducted.

First, a one-way analysis of variance (ANOVA) was used to investigate whether advertisements price–no price versus low price versus high price-, and, brand–brand versus generic–information, led to different purchase intentions between groups. The main objective of this empirical study was to explore the effect of different OTC advertisements on consumers’ purchase intention.

To examine the influence and predictive value of all variables (price and brand information, perceived risk and value, etc.) on consumers’ purchase intention, a regression analysis was run. It allowed to determine the overall variance explained by the predictors (fit of the model) and their relative contribution [37]. Only data from groups that were presented with prices were further investigated. Prior to the regression analysis, correlations between the various variables and purchase intention were tested using Pearson’s [50] correlations [37].

Subsequently, two parallel mediation models, ***A*** and ***B,*** were proposed and tested based on Dodd et al.’s market cue evaluation model, using Hayes’ PROCESS Plugin for SPSS [51]. Mediation analysis is a popular means to examine mechanisms by which causal effects between an independent and a dependent variable operate [52]. Four conditions have to be met in order to confirm a total mediation model. Firstly, the regression of the dependent variable on the independent variable, ignoring the mediators, should be significant (*X* → *Y*). Secondly, the regressions of the mediators on the independent variable should be significant (*X* → *M*1 and *M*2). Thirdly, the regressions of the dependent variable on the mediators, controlling for the independent variable, should be significant (*M*|*X* → *Y*). Fourthly, the regression of the dependent variable on the independent variable, controlling for the mediators is non-significant and nearly zero (*X*|*M* → *Y*) [53]. PROCESS simplifies the implementation of mediation analysis with observed variables and calculates bootstrapping in a way that facilitates inference.

**Fig. 2:**
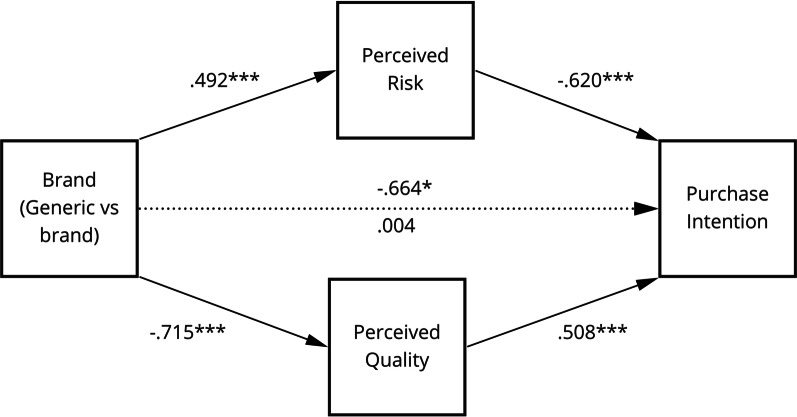
Model **A** depicting the relationship between brand information and purchase

**Fig. 3:**
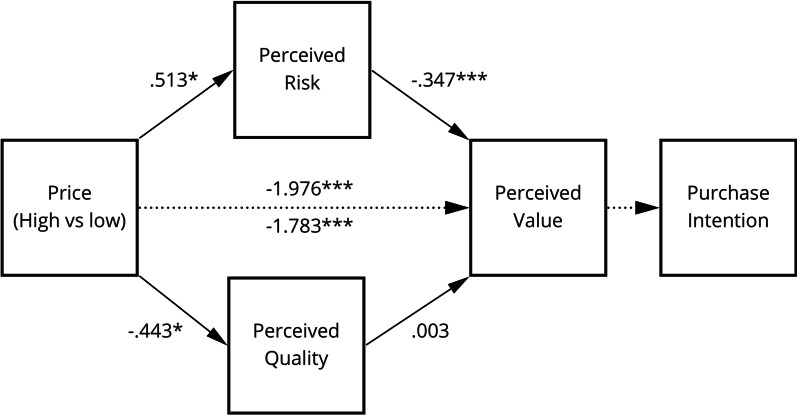
Model **B** depicting the relationship between price information and perceived value with perceived risk and perceived quality as mediators and purchase intention as the final outcome

Model ***A***, depicted in Fig. 2, explored whether perceived risk and perceived quality act as parallel mediators between brand information and purchase intention. Perceived value was not included in the model as this construct focused exclusively on participants’ perceptions of the presented price information. In line with Dodds’ conceptual framework [44], Model ***B ***examined the effect of price on perceived value, in the presence of two parallel mediators: perceived risk and perceived quality (cf. Fig. 3). Because perceived value is considered to be a direct antecedent of purchase intention [54], the last step of the conceptual model was not specifically examined. The results were organised according to Baron and Kenny’s causal steps approach [53].

### Open-ended qualitative data

Finally, a simplified version of Braun and Clarke’s thematic analysis was used to examine the surveys’ open-ended questions [55]. All written comments were read several times to become familiar with the data. During this process initial thoughts and ideas were noted down and recurrent features were coded. All responses were collated to different codes, which identified those features of the data that appeared interesting and meaningful to the question at hand. Codes were then clustered into themes.

## Results

In total 253 participants completed the online survey. Of those, 27.6% (*n* = 70) were considered to be incomplete due to missing responses. Additional file 1: Table S1 shows the distribution of all demographic sample characteristics, including age, sex, income, level of education, job and type of insurance. A sensitivity analysis showed no significant effects of the different demographic characteristics (gender, job, education, income, insurance etc.) on purchase intention. Reliability checks of the questionnaires revealed that all questionnaires showed adequate internal consistency [56].

The analysis of variance showed significant difference between groups (*F*(5, 82.3) = 10.238, *p* < 0.001), with post-hoc tests revealing that participants were least likely to purchase high-priced generic as opposed to low and high-priced brand, as well as a brand without a price. Participants were also found to be more likely to purchase the low-priced generic and the low-priced brand than the generic without a price, suggesting that the display of low-price information may have had an effect on consumers’ purchase intention. All other comparisons were found to be insignificant. Hence, hypothesis 1a could not be confirmed while and hypothesis 1b could be confirmed. Descriptive and post-hoc tests can be found in Additional file 2: Tables S3 and S4.

Next, a multiple linear regression analysis was conducted to predict the dependent variable (purchase intention) from the predictor variables (price, brand, perceived quality etc.). Prior to regression analysis, correlations between the various variables and purchase intention were tested using Pearson’s correlation analysis. A summary of all correlation coefficients is displayed in Additional file 3: Table S5.

The regression analysis consisted of two steps. Because price and brand information were the main focus of the study, these were entered first into the model. Then a forced entry method (one step) was applied to calculate the predictive value of all other variables. The results of the first multiple regression indicated that the two predictors, price and brand, explained 17.4% of the variance (adjusted* R*^2^ = 0.160, *F*(2,119) = 12.562, *p* < 0.001), indicative for a moderate goodness-of-fit according to Cohen. It was found that price significantly predicted purchase intention (*β* = − 0.375, *t*(119) =  − 4.502, *p* < 0.001), as did brand (*β* = − 0.188, *t*(119) =  − 2.261, *p* < 0.05), confirming hypothesis 2.

The second multiple regression analysis including all possible predictors (cf. Additional file 4: Table S6) and was also found to be significant (*F*(9,112) = 17.832, *p* < 0.001) and explained 58,9% of variance (adjusted *R*^2^ = 0.566).

Results indicate a 41.5% increase in explanatory power compared to the first model. Three variables significantly predicted purchase intention, perceived quality (*β* = 0.252, *t*(112) = 2.983, *p* < 0.01), perceived risk (*β* = − 0.285, *t*(112) = − 3.346, *p* < 0.001) and perceived value (*β* = 0.498, *t*(112) = 5.048, *p* < 0.001).

### Mediation analysis

Two mediation models, ***A*** and ***B,*** were proposed and tested. Model* A* (cf. Fig. 2), explored whether perceived risk and perceived quality act as parallel mediators between brand information and purchase intention. Model ***B ***examined the effect of price on perceived value, in the presence of the parallel mediators: perceived risk and perceived quality (cf. Fig. 3).

Step one of mediation Model ***A*** showed that brand information significantly predicted participants’ purchase intention, ignoring both mediators (i.e. perceived quality and perceived risk), *b* = − 0.664, *t*(120) =  − 2.031, *p* < 0.05 (*β* = − 0.363). The second step indicated that the regression of brand information on both mediators was also significant, for perceived risk *b* = 0.492, *t*(120) = 2.080, *p* < 0.05 (*β* = 0.371) and for perceived quality *b* = − 0.715, *t*(120) =  − 3.941, *p* < 0.001 (*β* = − 0.674). The third step of the mediation process confirmed the relationship between both mediators and purchase intention, controlling for brand information, with perceived risk *b* = − 0.620, *t*(118) =  − 5.328, *p* < 0.001 (*β* = − 0.449) and perceived quality *b* = 0.508, *t*(118) = 3.346, *p* < 0.001 (*β* = 0.294). Lastly, step four of the analysis revealed that, controlling for both mediators, brand information was not a significant predictor of purchase intention, *b* = 0.004, *t*(118) = 0.015, *p* = 0.988 (*β* = 0.002). Thus, perceived quality and perceived risk fully mediated the relationship between brand information and purchase intention, confirming hypothesis 3.

The analysis of Model ***A*** reveals that brand information indirectly impacts purchase intention through both perceived risk (indirect effect: − 0.305; 95% CI: − 0.666 to − 0.017) as well as perceived quality (indirect effect: − 0.363; 95% CI: − 0.710 to − 0.106). The mediations coexist in parallel and do not differ significantly in size (difference: 0.058; 95% CI: − 0.406 to 0.493), suggesting neither of the mediators play a greater role in explaining the effect of brand information on purchase intention. The model reveals that the branded drug increases perceptions of quality whilst decreasing levels of risk.

Step one of mediation Model ***B*** indicated that price information significantly predicted perceived value, ignoring both mediators (perceived quality and perceived risk), *b* = − 1.976, *t*(120) =  − 10.833, *p* < 0.001 (*β* = − 0.703*)*. The second step revealed that the regression of price information on both mediators was also significant, for perceived risk *b* = 0.513 *t*(120) = 2.174, *p* < 0.05 (*β* = 0.195) and for perceived quality *b* = − 0.443, *t*(120) = − 2.349, *p* < 0.05 (*β* = − 0.210*)*. However, the third step of the mediation process only confirmed the relationship between perceived risk and perceived value, controlling for price information, *b* = − 0.347, *t*(118) = − 4.595 *p* < 0.001 (*β* = − 0.326), suggesting that perceived risk might acted as a mediator whereas perceived quality did not, *b* = 0.033, *t*(118) = 0.350, *p* = 0.727 (*β* = 0.025). Finally, step four of the analysis showed that, controlling for both mediators, price information was still a significant predictor of perceived value, *b* = − 1.783, *t*(118) = − 10.689, *p* < 0.001 (*β* = − 0.634).

Thus, perceived quality and perceived risk did not fully mediate the relationship between price information and perceived value, only partly confirming hypothesis 4. The results suggest that perceived risk partially mediated the effect of price information on perceived value (indirect effect: − 0.178; 95% CI: − 0.426 to − 0.013). The results indicate that low price increases perceived quality and decreases perceived risk.

**Fig. 4:**
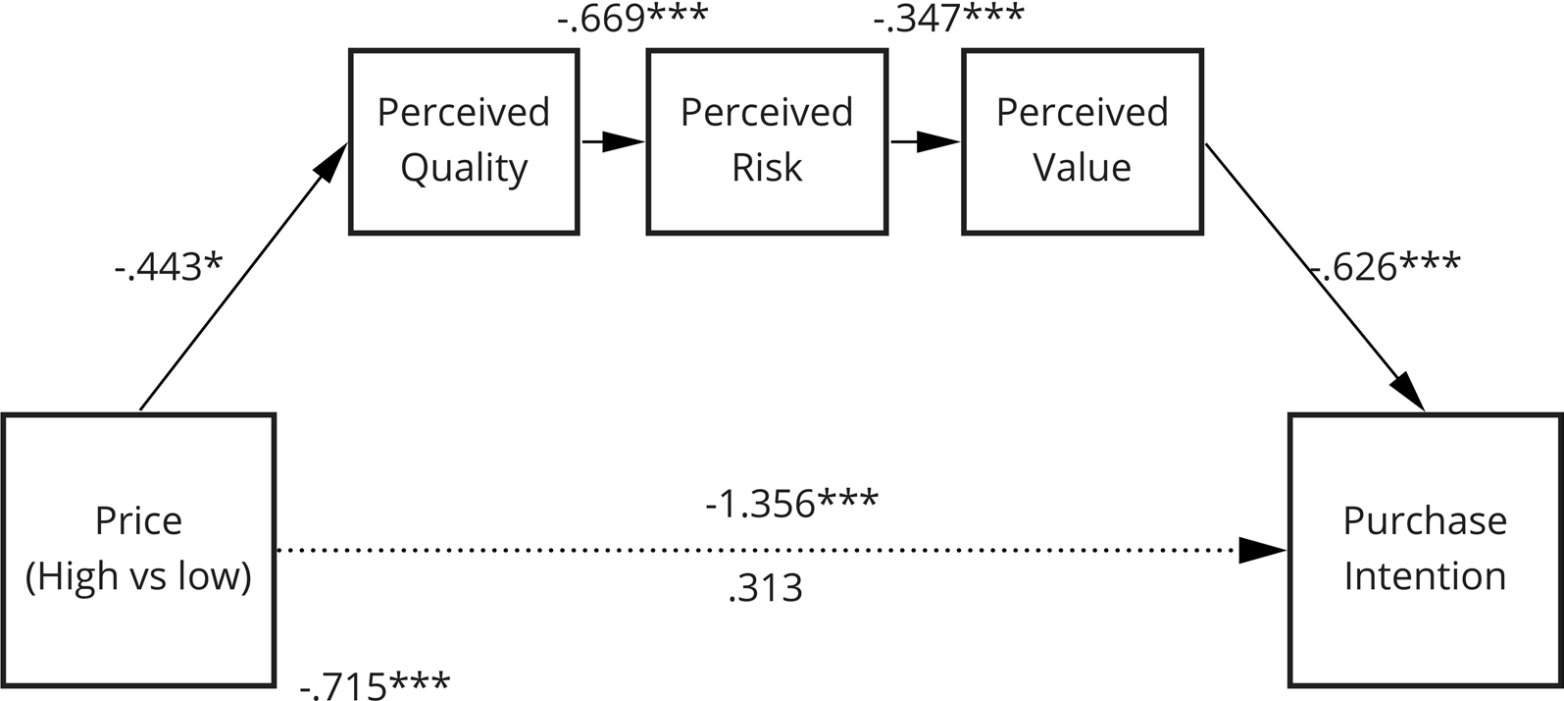
Model **C** depicting the relationship between price information and purchase intention with perceived quality, perceived risk and perceived value as serial mediators

In light of the previously outlined multiple regression and correlation analysis, as well as findings from Model **B**, we tested for a serial mediation to predict purchase intention from price. Therefore, a new Model **C** was proposed**,** which represents a serial mediation between price and purchase intention through perceived quality, perceived risk and perceived value, as depicted in Fig. 4.

All mediators predicted purchase intention whilst controlling for price information (perceived quality: *b* = 0.427, *t*(117) = 3,369 (*β* = 0.248*)*, *p* < 0.001; perceived risk: *b* = − 0.367, *t*(117) = − 3.339, *p* < 0.001 (*β* = − 0.265*)*; perceived value: *b* = 0.626, *t*(117) = 5.087, *p* < 0.001 (*β* = 0.483). However, controlling for all three mediators, the regression of price information on purchase intention was no longer significant (*b* = 259, *t*(117) = 0.828 *p* = 0.409; *β* = 0.142), suggesting that mediation had taken place.

Findings indicated that the mediation of price information through perceived quality via perceived risk and perceived value to purchase intention was significant (indirect effect: − 0.067; 95% CI: − 0.176 to − 0.011), as was the indirect path through perceived quality (indirect effect: − 0.189; 95% CI: − 0.418 to − 0.025) and perceived value (indirect effect: − 1.117; 95% CI: − 1.719 to − 0.616). In contrast, the indirect effect of price information through perceived risk was insignificant (95% CI: − 0.222 to 0.088) and so where all other indirect paths between the variables. The findings show that low prices positively affected perceived quality. Taken together, the data support the serial mediation hypothesis: as price decreases perceived quality of the OTC drug increases, this then decreases perceived risk, which in turn increases perceived value and ultimately affects purchase intent. In addition, perceived risk does not act as an independent mediator but rather is part of a longer causal chain.

### Open-ended question

To gain deeper insight about whether consumers thought that the presentation of prices in OTC advertisements the surveys’ open-ended data emerged into the following three themes (cf. Additional file 5: Table S7): (1) Unawareness and misconceptions; (2) past experience; and, (3) advertising as a decision-making tool*.*

1. *Unawareness and misconceptions*
  Participants showed limited awareness regarding the comparability of generics and branded OTC drugs and expressed concerns regarding the efficacy and safety of generic alternatives. Consequently, price information in advertising appeared to play a minor role in the purchase intention of consumers that have negative perceptions about generic OTC drugs. There was also evidence that this understanding might result from lacking information about the benefits and bioequivalence of generic alternatives, suggesting that increased awareness may alter consumers’ purchase intention.
2. *Past experience*
  Participants stated to be more likely to buy a branded drug that has been on the market for years than a newer and less advertised generic alternative, regardless of the price. Thus, past experiences with a branded or a generic drug appeared to have a decisive impact on consumers’ purchase intention with health professionals as important initiators of OTC drug use.
3. *Advertising as a decision-making tool*
  Participants expressed mixed views about the value of drug advertisements. While some participants perceived OTC drug advertisements as important and helpful, others believed that they are inauthentic and untrustworthy. Overall, however, advertisements were assumed to increase transparency and competition with some respondents explicitly questioning the association between higher prices and better quality for OTC drugs.

## Discussion

Most industrialised countries have long recognised the importance of promoting responsible self-medication as a critical building block for effective and efficient healthcare; however, the market diffusion has been slow. The focus of the present study was to closely examine the relationship between price and brand information and consumers’ purchase intention. For this, a modified version of the market cue–product evaluation model by Dodds et al. [26] was tested to uncover the main processes that underlie consumers’ purchasing behaviour in response to OTC advertising.

Overall, the results showed that participants were least likely to purchase a high-priced generic as opposed to low and high-priced brand, as well as a brand without a price. Participants were also found to be more likely to purchase the low-priced generic and the low-priced brand than the generic without a price, suggesting that the display of low-price information may have had an effect on consumers’ purchase intention. Hence, hypothesis 1 was partly confirmed; while there was no difference in increased purchase intention for branded OTC drug compared to the generic OTC drug when no price is presented (brand effect: H 1a), participants were more likely to purchase low-priced generic OTC drug compared to the high-priced branded drug when the price was reported (price awareness: H 1b).

Findings also provide critical insights into the processes and factors that underlie consumer purchasing patterns of OTC drugs (cf. hypotheses 2–4). It was shown that price and brand information significantly predicted participants’ purchase intention in the absence of other potential predictors, explaining 17.4% of the variance, confirming hypothesis 2. Adding other predictive variables showed a 41.5% increase in explanatory power. Three variables significantly predicted purchase intention, including perceived quality, perceived risk, and perceived value.

It was, furthermore, demonstrated that the relationship between price/brand information and participants’ purchase intention was significantly mediated by perceived risk and quality (i.e. Model **A**). While the brand information led to a decreased perception of risk and increased perception of quality, the opposite was found for the display of a generic drug. In other words, perceived quality and perceived risk fully mediated the relationship between brand information and purchase intention, confirming hypotheses 3(a–c). These findings are in agreement with previous research [35, 57], showing that well-known brands act as a cue for increased quality and reduced risk.

In relation to hypothesis 4, findings showed that perceived quality and perceived risk did not fully mediate the relationship between price information and perceived value, which, in turn, is expected to predict purchase intention (i.e. Model **B**). While there was a significant inverse relationship between perceived risk and perceived value (cf. H4(c)), there was none for perceived quality and perceived value (cf. H4b). The exposure to high-priced drug advertisements did not increase perceptions of quality and decrease perceptions of risk (cf. H4(a)); instead low price increased perceived quality and decreases perceived risk. At first glance these results appear counterintuitive; however, recent research has shown that cost savings gained from the use of generic prescription drugs can create a positive appeal to consumers who had previous experience with generics [58]. This appeal possibly bridges the price-perceived quality gap, explaining its reverse notion found in both our quantitative and qualitative data (e.g. ‘Good quality and a higher price are sometimes congruent, but not in this case’).

Because of the findings from our regression analysis and Model **B**, a third model was tested (Model **C**), where perceived value, risk and quality acted as part of a serial mediation. Results confirmed that price information and purchase intentions were predicted through consumers’ perceptions of quality, risk and value, and that perceived risk had an inverse relationship with perceived value and perceived quality. In other words, higher perceived quality reduced perceived risk, which, in turn, increased perceived value and purchase intention. While a link between price and brand information, as well as between perceived risk and perceived quality was established in previous research [18, 59, 60], there has been no evidence in relation to the influence of perceived value. This is a novel finding, showing that the evaluation of OTC drugs is not only based on the quality and associated risk, but also their value for money.

The simplified thematic analysis demonstrated that consumers perceived the display of prices and OTC advertising as a trigger to gather more information about medications, and that past experiences with either a generic or branded drug had an important effect on consumers’ purchase intention. They also highlighted that they would rely on pharmacists’ recommendations or seek their advice when purchasing an OTC drug, transforming the purchase into an active act of decision making. Overall, it appears that having awareness about different OTC drugs and their benefits becomes embedded in consumers’ decision-making process, making individuals more open to the influence of external cues such as price information in advertising. Since existing research demonstrated that price cues are particularly effective in promoting products to those consumers that have more knowledge about a product category [61, 62], current participants’ greater reliance on price as a promotional cue appears reasonable. This suggestion was further supported by participants comments. Thus, findings from the qualitative data extend current survey results, showing that the purchase intention is impacted by past experiences and attitudes towards advertisements as trustworthy/untrustworthy, and, furthermore, modifiable by a greater understanding of the differences and similarities between OTC drugs, communicated by a person of credibility (e.g. pharmacist).

### Policy and strategic implications

From a policy and commercial standpoint, it has several noteworthy implications for marketers and public policy makers.

In terms of pharmaceutical marketing, advertisement of actual prices may not be sufficient to incentivise consumers to switch from a branded OTC drug to a generic one. Yet, price advertising campaigns appear to contribute towards the broader objective of creating awareness of generic drugs’ (economic) benefits among consumers [63]. This growing awareness through advertising and educational campaigns appears to encourage purchase, and, if expanded, may facilitate market diffusion of generics.

Promotional and educational efforts should be targeted specifically at mitigating consumers’ perceptions of risk while improving their trust and confidence in the quality of generic drugs [57]. The communication of more objective information about generic drugs, including carefully selected (intrinsic) characteristics, may reflect a valuable means to educate consumers about their quality and comparability to national brands, thereby reducing perceptions of risk [63]. Policymakers may, therefore, wish to develop nationwide campaigns directed towards various stakeholders, including health professionals and the general public. These should embrace the use of generic medication as a whole and build a more favourable product image.

The qualitative data analysis also highlighted the need for policymakers to make use of the direct contact between consumers and pharmacists (or physicians) as a key communication channel. In Germany, pharmacies remain the first port of call when purchasing OTC drugs, and have considerable potential to influence consumer beliefs about generic drugs and their use. For this, it is important that measures are taken to ensure that this cohort is appropriately trained and willing to inform consumers about generic medications, in order to help broaden consumers’ acceptance of generic alternatives [64].

### Limitations and future research

The present study did not assess whether participants had a medical background or more advanced levels of knowledge about generic medicines prior to the experiment. Health professionals, pharmacists and other more knowledgeable individuals commonly hold more favourable attitudes towards generics and are, thus, more likely to purchase generic alternatives [20].

The artificial nature of the experiment may have limited the study’s external validity and have prompted behaviour that is absent under more realistic conditions [65]. Despite the fact that the design of all advertisements in the present study was conceptually close to original drug advertising and was validated by experts in the field of pharmaceutical marketing, future research should investigate the impact of advertising in real consumer environments.

Concomitantly, it is important to note that the study relied on consumers’ self-reported purchase intention rather than on actual sales data. Thus, the study had to rely on the participants’ integrity to respond honestly to the questionnaires. While observational studies are a better means to observe participants’ actual choices [66], and future studies are encouraged to do so, this endeavour would have exceeded the purpose of the present experiment.

Our findings are limited to print media; contemporary marketing, however, utilises a broad range of communication channels and formats to promote medicines, such as television, social media and drug packaging. Different channels might reach different target groups, e.g. the older generation, which is less well represented in the present study. Investigating the synergistic and different effects of these various advertisement approaches would likely increase the generalisability of the present results and provide insights into the most powerful way to promote the use of generic OTC drugs.

Finally, the current study only addressed one therapeutic area of OTC drugs: pain medicines. Although these drugs are the most frequently consumed type of OTC drug, potentially varying perceptions associated with different therapeutic areas should be explored. Additionally, scholars may wish to investigate the role of different health-related consumer characteristics.

## Conclusion

Increasingly more European countries struggle with rising healthcare costs and the assurance of a sustainable healthcare system in the face of mounting demographic pressures and increasing levels of public expenditure. The promotion of generic OTC drugs for self-medication purposes is considered to represent an effective means to tackle these challenges. Two main recommendations emerge from these conclusions: (1) authorities should allocate budgets and invest in interventions to educate consumers and healthcare professionals about the bioequivalence of different available OTC drugs; and, (2) effective extrinsic marketing cues should be posited along with intrinsic product cues, such as the active ingredient of a drug, for products to occupy a meaningful and distinct competitive position in consumer’s purchase environment.

## Supplementary Information

**Additional file 1: Table S2.** Participants’ characteristics (N=183).

**Additional file 2: Table S3.** Mean and standard deviations of purchase intention by advertisement group. **Table S4.** Post hoc results for purchase intention by advertisement group.

**Additional file 3: Table S5.** Correlation coefficients between study variables.

**Additional file 4: Table S6. **Predictors of purchase intention of OTC drugs.

**Additional file 5: Table S7.** Examples of comments made by participants in response to the open-ended question.

## Data Availability

The data that support the findings of this study are not publicly available. Because of the nature of the informed consent and ethical restrictions, data distribution is not permitted.

## Abbreviations

OTC: Over the counter
DTC: Direct to consumer
ANOVA: Analysis of variance
