# Supplementary material for: The risk-value trade-off: price and brand information impact consumers’ intentions to purchase OTC drugs
Source: J Pharm Policy Pract. 2021 Jan 25;14:11. doi: 10.1186/s40545-020-00293-5 (PMC7831199; doi:10.1186/s40545-020-00293-5)
Supplement: Supplementary file 1 — Additional file 1: Table S2. Participants’ characteristics (N=183). [file 40545_2020_293_MOESM1_ESM.docx]

| Table 2  Participants’ characteristics (N=183). |  |
| --- | --- |
|  | Summary measures |
| **Age** (mean, range) | *M*=39.5 years (18 — 83) |
| **Sex** (frequencies)  Female  Male | 119 (65%)  64 (35%) |
| **Education**  Do not want to say  Less than High School  Apprenticeship (without university degree)  Bachelor’s degree  Master’s degree  PhD | 12 (6,6%)  5 (2,7%)  49 (26,8%)  48 (26,2%)  60 (32,8%)  9 (4,9%) |
| **Income** (year)  Do not want to say  less than 25.000€  25.000€ to 50.000€  50.000€ to 75.000€  75.000€ to 125.000€  more than 125.000€ | 13 (7,1%)  38 (20,8%)  51 (27,9%)  60 (33,8%)  16 (8,7%)  5 (2,7%) |
| **Job**  Untrained Workforce  Skilled Workforce  Self-employed  Office Workforce  Lower Management  Higher Management | 8 (4,4%)  6 (3,3%)  14 (7,7)  108 (59%)  43 (23,5%)  4 (2,2%) |
| **Insurance**  Public health insurance  Private health insurance | 127 (69,4%)  56 (30,6%) |
