# Supplementary material for: The risk-value trade-off: price and brand information impact consumers’ intentions to purchase OTC drugs
Source: J Pharm Policy Pract. 2021 Jan 25;14:11. doi: 10.1186/s40545-020-00293-5 (PMC7831199; doi:10.1186/s40545-020-00293-5)
Supplement: Supplementary file 2 — Additional file 2: Table S3. Mean and standard deviations of purchase intention by advertisement group. Table S4. Post hoc results for purchase intention by advertisement group. [file 40545_2020_293_MOESM2_ESM.docx]

| Table 3  Mean and standard deviations of purchase intention by advertisement group. | | |
| --- | --- | --- |
|  | *N* | *M (SD)* |
| Brand without Price  Generic without Price  Brand high Price  Generic high Price  Brand low Price  Generic low Price | 31  30  30  29  31  32 | 4.25 (1.99)  3.52 (1.44)  4.25 (1.59)  2.76 (1.11)  4.84 (1.63)  4.91 (2.04) |

| Table 4  Post hoc results for purchase intention by advertisement group. | | | | | | | |
| --- | --- | --- | --- | --- | --- | --- | --- |
|  | *Mean* |  | Mean Differences (Xi −X j) | | | |  |
|  |  | 1 | 2 | 3 | 4 | 5 | 6 |
| 1. Brand without Price  2. Generic without Price  3. Brand high Price  4. Generic high Price  5. Brand low Price  6. Generic low Price | 4.25  3.52  4.25  2.76  4.84  4.91 | —  .73  .00  1.49**  -.59  -.66 | —  -.73  .77  -1.31*  -1.38* | —  1.49***  -.59  -.66 | —  -2.08***  -2.15*** | —  -.07 | — |
| Note. *N*=183. **p*<.05, ** *p*<.01; ****p*<.001 | | | | | |  |  |
