# Supplementary material for: The risk-value trade-off: price and brand information impact consumers’ intentions to purchase OTC drugs
Source: J Pharm Policy Pract. 2021 Jan 25;14:11. doi: 10.1186/s40545-020-00293-5 (PMC7831199; doi:10.1186/s40545-020-00293-5)
Supplement: Supplementary file 3 — Additional file 3: Table S5. Correlation coefficients between study variables. [file 40545_2020_293_MOESM3_ESM.docx]

| Table 5  Correlation coefficients between study variables. | | | | | | |  |  |  |  |
| --- | --- | --- | --- | --- | --- | --- | --- | --- | --- | --- |
| Variable | 1 | 2 | 3 | 4 | 5 | 6 | 7 | 8 | 9 | 10 |
| 1. PI | — |  |  |  |  |  |  |  |  |  |
| 2. Price | -.372*** | — |  |  |  |  |  |  |  |  |
| 3. Brand | -.182* | -.016 | — |  |  |  |  |  |  |  |
| 4. PQ | .552*** | -.210* | -.339*** | — |  |  |  |  |  |  |
| 5. PR | -.618*** | .195* | .187* | -.575*** | — |  |  |  |  |  |
| 6. PV | .642*** | -.703*** | -.025 | .346*** | -.464*** | — |  |  |  |  |
| 7. BL | -.182* | .102 | -.027 | -.235** | .217** | -.127 | — |  |  |  |
| 8. ATTG | -.276** | .142 | -.004 | -.346*** | .400*** | -.242** | .666*** | — |  |  |
| 9. PC | .106 | -.013 | .083 | .219* | -.192* | .030 | -.562*** | -.444*** | — |  |
| 10. EFF | .408*** | -.203 | -.233 | .525*** | -.496*** | .390*** | -.007 | -.166 | .051 | — |
| *p* < .05. ***p* < .01. ****p* < .001. *Note*. *N*=122. PI= Purchase intention, PQ = Perceived Quality, PR= Perceived Risk, PV = Perceived Value, BL = Brand Loyalty, ATTG = Attitude towards generics (negative), PC = Price Consciousness, EFF = Efficacy | | | | | | | | | | |
