# Supplementary material for: The risk-value trade-off: price and brand information impact consumers’ intentions to purchase OTC drugs
Source: J Pharm Policy Pract. 2021 Jan 25;14:11. doi: 10.1186/s40545-020-00293-5 (PMC7831199; doi:10.1186/s40545-020-00293-5)
Supplement: Supplementary file 4 — Additional file 4: Table S6. Predictors of purchase intention of OTC drugs. [file 40545_2020_293_MOESM4_ESM.docx]

| Table 6  Predictors of purchase intention of OTC drugs. | | |  |
| --- | --- | --- | --- |
|  | *B* | *SE B* | *β* |
| ***Step 1*** |  |  |  |
| Constant  Brand  Price | 6.589  -1.367  -.686 | .501  .304  .304 | -.375***  -.188* |
| ***Step 2*** |  |  |  |
| Constant  Price  Brand  Perceived Quality  Perceived Risk  Perceived Value  Brand Loyalty  Attitude towards Generics  Price Consciousness  Efficacy | .613  .270  -.148  .434  -.394  .646  -.071  .066  -.024  -.077 | 1.572  .319  .241  .145  .118  .128  .121  .106  .103  .132 | .074  -.041  .252**  -.285***  .498***  -.053  .054  -.017  -.045 |
| Note. *N*=122. **p*<.05, ** *p*<.01; ****p*<.001. Brand was coded as 0=branded drug and 1=generic drug while price is coded as high=2 and low=1. | | |  |
