# Supplementary material for: The risk-value trade-off: price and brand information impact consumers’ intentions to purchase OTC drugs
Source: J Pharm Policy Pract. 2021 Jan 25;14:11. doi: 10.1186/s40545-020-00293-5 (PMC7831199; doi:10.1186/s40545-020-00293-5)
Supplement: Supplementary file 5 — Additional file 5: Table S7. Examples of comments made by participants in response to the open-ended question. [file 40545_2020_293_MOESM5_ESM.docx]

| Table 7  Examples of comments made by participants in response to the open-ended question. | | |
| --- | --- | --- |
| Theme | Nr. | Quote |
| Unawareness and Misconceptions | 1.  2.  3.  4.  5.  6.  7. | ‘I am not really convinced of the equal effect of generics and would, therefore, always buy a branded product’  ‘When it comes to medicines, I always buy branded products. Generics seem too unsafe’  ‘Price is not so relevant for OTC medications, I only trust branded products.’  ‘I think most Germans have prejudices against generics, if you break these down prices could play an important role’  ‘[People’s] awareness regarding the equal effect of generic and branded products needs to be strengthened’  ‘If generics are as effective as [branded OTC drugs] but are advertised with an attractive price, I would choose the generic.’  ‘I now often use generic drugs because my pharmacist has made me aware.’ |
| Past experience | 8.  9.  10.  11. | ‘[Prices do not influence my purchase intention] I only buy things that I know or that my doctor recommends to me’  ‘I prefer to rely on experience reports’  ‘It depends on whether I already had experience with the product (if I know that the generic drug is good, then I would buy it because it is cheaper).. Maybe if pharmacists would draw my attention to the price’  ‘I always ask the pharmacist for the price, ask for recommendations and educate myself about the individual products’ |
| Advertising as a decision-making tool | 12.  13.  14.  15.  16.  17.  18. | ‘Advertising is an important decision-making factor, so I think greater advertising of generics could increase their popularity’    ‘I generally perceive drug advertising as questionable and untrustworthy’  ‘Prices are not really displayed in the pharmacy, thus prices in advertising would help consumers to make a decision’    ‘[Price advertisements] allow for direct comparability’  ‘Good quality and a higher price are sometimes congruent, but not in this case’  ‘Especially people who pay attention to their money would use price information - I myself use online comparison portals’  ‘It could trigger the visit to the pharmacy, where the use of a generic maybe recommended’ |
